# 9-(2-Bromo­phen­oxy­carbon­yl)-10-methyl­acridinium trifluoro­methane­sulfonate

**DOI:** 10.1107/S1600536812020892

**Published:** 2012-05-16

**Authors:** Damian Trzybiński, Andrzej Sieradzan, Karol Krzymiński, Jerzy Błażejowski

**Affiliations:** a Faculty of Chemistry, University of Gdańsk, J. Sobieskiego 18, 80-952 Gdańsk, Poland

## Abstract

In the crystal structure of the title compound, C_21_H_15_BrNO_2_^+^·CF_3_SO_3_^−^, adjacent cations are linked through C—Br⋯π and π–π contacts [centroid–centroid distance = 3.744 (2) Å], and neighbouring cations and anions *via* C—H⋯O, C—F⋯π and S—O⋯π inter­actions. The acridine and benzene ring systems are oriented at a dihedral angle of 18.7 (1)°. The carb­oxy group is twisted at an angle of 69.3 (1)° relative to the acridine skeleton. The mean planes of adjacent acridine moieties are either parallel or inclined at an angle of 27.8 (1)° in the lattice.

## Related literature

For general background to the chemiluminescent properties of 9-phen­oxy­carbonyl-10-methyl­acridinium trifluoro­methane­sulfonates, see: King *et al.* (2007 ▶); Krzymiński *et al.* (2011 ▶); Roda *et al.* (2003 ▶); Zomer & Jacquemijns (2001 ▶). For related structures, see: Trzybiński *et al.* (2010 ▶). For inter­molecular inter­actions, see: Dorn *et al.* (2005 ▶); Hunter *et al.* (2001 ▶); Novoa *et al.* (2006 ▶); Seo *et al.* (2009 ▶); Sikorski *et al.* (2005 ▶); Trzybiński *et al.* (2010 ▶). For similar C–Br⋯π, π–π, C–H⋯O, C–F⋯π and S–O⋯π inter­actions in related compounds, see: Sikorski *et al.* (2005 ▶); Trzybiński *et al.* (2010 ▶). For the synthesis, see: Sato (1996 ▶); Trzybiński *et al.* (2010 ▶).
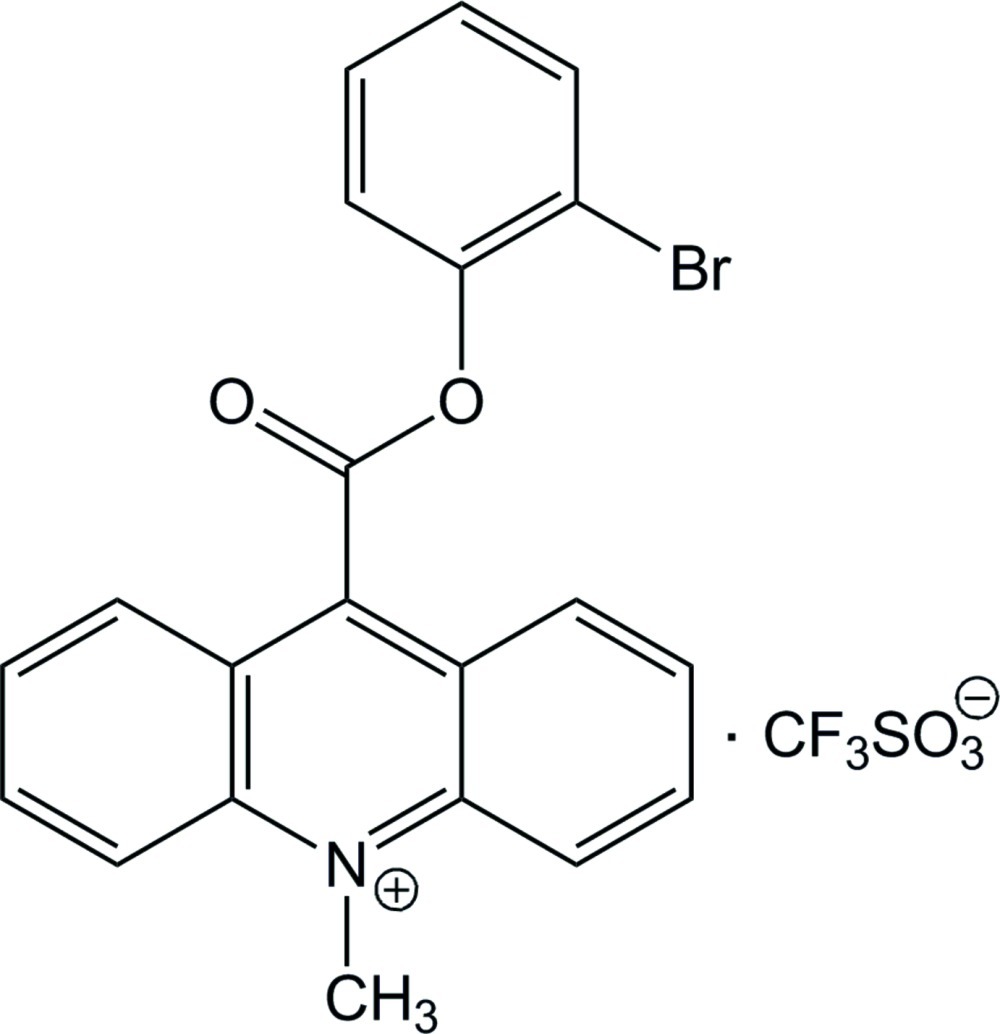


## Experimental

### 

#### Crystal data

C_21_H_15_BrNO_2_^+^·CF_3_O_3_S^−^
*M* *_r_* = 542.32
Monoclinic, 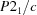

*a* = 12.5718 (8) Å
*b* = 20.3617 (16) Å
*c* = 8.5162 (6) Å
β = 104.498 (7)°
*V* = 2110.6 (3) Å^3^
*Z* = 4
Mo *K*α radiation
μ = 2.11 mm^−1^
*T* = 295 K
0.46 × 0.25 × 0.02 mm

#### Data collection

Oxford Diffraction Gemini R Ultra Ruby CCD diffractometer
Absorption correction: multi-scan (*CrysAlis RED*; Oxford Diffraction, 2008 ▶) *T*_min_ = 0.668, *T*_max_ = 1.000
16372 measured reflections
3735 independent reflections
2348 reflections with *I* > 2σ(*I*)
*R*_int_ = 0.041

#### Refinement

*R*[*F* ^2^ > 2σ(*F* ^2^)] = 0.035
*wR*(*F* ^2^) = 0.088
*S* = 0.92
3735 reflections
299 parameters
H-atom parameters constrained
Δρ_max_ = 0.46 e Å^−3^
Δρ_min_ = −0.40 e Å^−3^

### 

Data collection: *CrysAlis CCD* (Oxford Diffraction, 2008 ▶); cell refinement: *CrysAlis RED* (Oxford Diffraction, 2008 ▶); data reduction: *CrysAlis RED*; program(s) used to solve structure: *SHELXS97* (Sheldrick, 2008 ▶); program(s) used to refine structure: *SHELXL97* (Sheldrick, 2008 ▶); molecular graphics: *ORTEP-3* (Farrugia, 1997 ▶); software used to prepare material for publication: *SHELXL97* and *PLATON* (Spek, 2009 ▶).

## Supplementary Material

Crystal structure: contains datablock(s) global, I. DOI: 10.1107/S1600536812020892/fj2548sup1.cif

Structure factors: contains datablock(s) I. DOI: 10.1107/S1600536812020892/fj2548Isup2.hkl

Supplementary material file. DOI: 10.1107/S1600536812020892/fj2548Isup3.cml

Additional supplementary materials:  crystallographic information; 3D view; checkCIF report

## Figures and Tables

**Table 1:** Hydrogen-bond geometry (Å, °)

*D*—H⋯*A*	*D*—H	H⋯*A*	*D*⋯*A*	*D*—H⋯*A*
C3—H3⋯O28^i^	0.93	2.54	3.289 (5)	137
C7—H7⋯O27	0.93	2.54	3.200 (5)	128

Symmetry code: (i) 
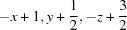
.

**Table 2:** C—F⋯π, C—Br⋯π and S—O⋯π inter­actions (Å, °) *Cg*1, *Cg*2 and *Cg*4 are the centroids of the C9/N10/C11–C14, C1–C4/C11/C12 and C18–C23 rings, respectively.

*X*—*I*⋯*J*	*I*⋯*J*	*X*⋯*J*	*X*—*I*⋯*J*
C19—Br24⋯*Cg*4^ii^	3.523 (2)	4.847 (3)	124.6 (1)
C30—F32⋯*Cg*4^iii^	3.648 (2)	4.310 (4)	110.8 (2)
S26—O27⋯*Cg*1^iv^	3.821 (3)	3.708 (2)	74.8 (2)
S26—O28⋯*Cg*1^iv^	3.414 (3)	3.708 (2)	90.3 (2)
S26—O28⋯*Cg*2^iv^	3.358 (3)	4.445 (2)	132.2 (2)

Symmetry codes: (ii) 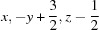; (iii) 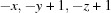; (iv) 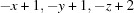.

**Fig. 1.**
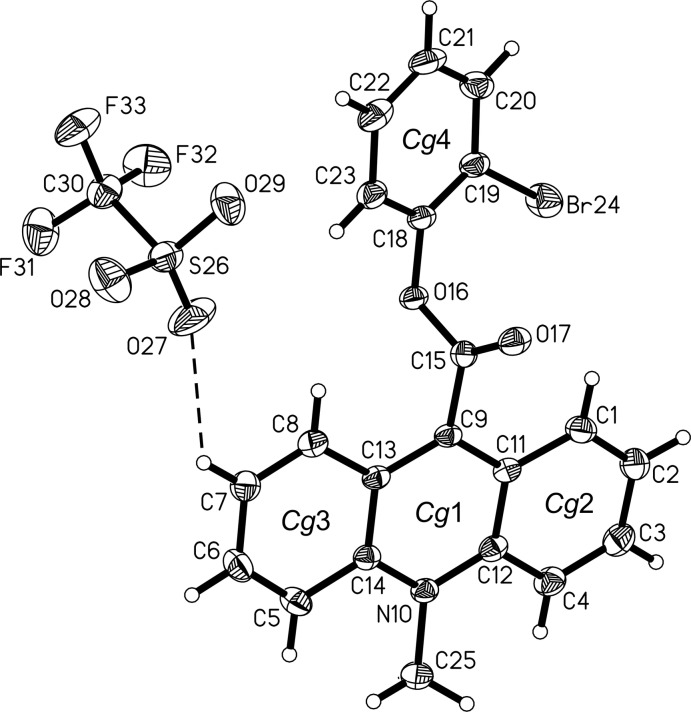
The molecular structure of the title compound showing the atom-labeling scheme. Displacement ellipsoids are drawn at the 25% probability level and H atoms are shown as small spheres of arbitrary radius. Cg1, Cg2, Cg3 and Cg4 denote the ring centroids. The C–H···O hydrogen bond is represented by a dashed line.

**Fig. 2.**
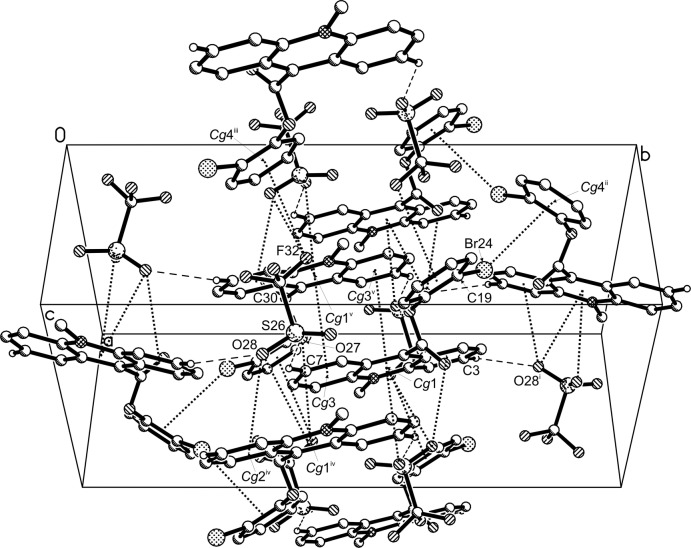
The arrangement of the ions in the crystal structure. The C–H···O interactions are represented by dashed lines, the C–F···π, C–Br···π, S–O···π and π–π contacts by dotted lines. H atoms not involved in interactions have been omitted. [Symmetry codes: (i) –x + 1, y + 1/2, –z + 3/2; (ii) x, –y + 3/2, z – 1/2; (iii) –x, –y + 1, –z + 1; (iv) –x + 1, –y + 1, –z + 2; (v) –x + 1, –y + 1, –z + 1.]

## supplementary crystallographic information

### Comment

The well-known chemiluminescence of 9-(phenoxycarbonyl)-10-methylacridinium salts has been utilized in chemiluminescent indicators and labels, which are commonly applied in assays of biologically and environmentally important entities (Zomer & Jacquemijns, 2001; Roda *et al.*, 2003; King *et al.*, 2007). The cations of these salts are oxidized by H_2_O_2_ in alkaline media, a reaction that is accompanied by the removal of the phenoxycarbonyl fragment and the conversion of the remaining part of the molecules to electronically excited, light-emitting 10-methyl-9-acridinone (Krzymiński *et al.*, 2011). The efficiency of chemiluminescence – crucial for analytical applications – is affected by the constitution of the phenyl fragment (Zomer & Jacquemijns, 2001). In continuing our investigations on the latter aspect, we synthesized 9-(2-bromophenoxycarbonyl)-10-methylacridinium trifluoromethanesulfonate, whose crystal structure is presented here.

In the cation of the title compound (Fig. 1), the bond lengths and angles characterizing the geometry of the acridinium moiety are typical of acridine-based derivatives (Trzybiński *et al.*, 2010). With respective average deviations from planarity of 0.0519 (3) Å and 0.0034 (3) Å, the acridine and benzene ring systems are oriented at a dihedral angle of 18.7 (1)°. The carboxyl group is twisted at an angle of 69.3 (1)° relative to the acridine skeleton. The mean planes of the adjacent acridine moieties are parallel (remain at an angle of 0.0 (1)°) or inclined at an angle of 27.8 (1)° in the crystal.

The search for intermolecular interactions in the crystal using PLATON (Spek, 2009) has shown that the adjacent cations are linked by C–Br···π (Table 2, Fig. 2) and π-π (Table 3, Fig.2) contacts, and the cations and neighboring anions via C–H···O (Table 1, Figs. 1 and 2), C–F···π (Table 2, Fig. 2) and S–O···π (Table 2, Fig. 2) interactions. The C–H···O interactions are of the hydrogen bond type (Novoa *et al.* 2006). The C–F···π (Dorn *et al.*, 2005), S–O···π (Dorn *et al.*, 2005) and π–π (Hunter *et al.*, 2001) interactions should be of an attractive nature. The C–Br···π interactions have been reported by others (Seo *et al.*, 2009). We have found all the above interactions in many other 9-phenoxycarbonyl-10-methylacridinium trifluoromethanesulfonates (e.g. Sikorski *et al.*, 2005; Trzybiński *et al.*, 2010). Mentioning them here is important in the context of the analysis and understanding of the crystal architecture of this group of compounds. The crystal structure is stabilized by a network of these short-range specific interactions and by long-range electrostatic interactions between ions.

### Experimental

2-Bromophenylacridine-9-carboxylate was synthesized by esterification of 9-(chlorocarbonyl)acridine (obtained by treating acridine-9-carboxylic acid with a tenfold molar excess of thionyl chloride) with 2-bromophenol in anhydrous dichloromethane in the presence of N,N-diethylethanamine and a catalytic amount of N,N-dimethyl-4-pyridinamine (room temperature, 15h) (Sato, 1996). The product was purified chromatographically (SiO_2_, cyclohexane/ethyl acetate, 1/1 v/v) and subsequently quaternarized with a fivefold molar excess of methyl trifluoromethanesulfonate dissolved in anhydrous dichloromethane (Trzybiński *et al.*, 2010). The crude 9-(2-bromophenoxycarbonyl)-10-methylacridinium trifluoromethanesulfonate was dissolved in a small amount of ethanol, filtered and precipitated with a 20 v/v excess of diethyl ether. Light-orange crystals suitable for X-ray investigations were grown from methanol/water (2:1, v/v) solution (m.p. 495–497 K).

### Refinement

H atoms were positioned geometrically, with C–H = 0.93 Å and 0.96 Å for the aromatic and methyl H atoms, respectively, and constrained to ride on their parent atoms with *U*_iso_(H) = *xU*_eq_(C), where *x* = 1.2 for the aromatic and *x* = 1.5 for the methyl H atoms.

### Crystal data

**Table:**

C_21_H_15_BrNO_2_^+^·CF_3_O_3_S^−^	*F*(000) = 1088
*M**_r_* = 542.32	*D*_x_ = 1.707 Mg m^−^^3^
Monoclinic, *P*2_1_/*c*	Mo *K*α radiation, λ = 0.71073 Å
Hall symbol: -P 2ybc	Cell parameters from 1539 reflections
*a* = 12.5718 (8) Å	θ = 3.0–29.2°
*b* = 20.3617 (16) Å	µ = 2.11 mm^−^^1^
*c* = 8.5162 (6) Å	*T* = 295 K
β = 104.498 (7)°	Plate, light-orange
*V* = 2110.6 (3) Å^3^	0.46 × 0.25 × 0.02 mm
*Z* = 4	

### Data collection

**Table:**

Oxford Diffraction Gemini R Ultra Ruby CCD diffractometer	3735 independent reflections
Radiation source: Enhanced (Mo) X-ray Source	2348 reflections with *I* > 2σ(*I*)
Graphite monochromator	*R*_int_ = 0.041
Detector resolution: 10.4002 pixels mm^-1^	θ_max_ = 25.1°, θ_min_ = 3.2°
ω scans	*h* = −14→11
Absorption correction: multi-scan (*CrysAlis RED*; Oxford Diffraction, 2008)	*k* = −24→23
*T*_min_ = 0.668, *T*_max_ = 1.000	*l* = −10→10
16372 measured reflections	

### Refinement

**Table:**

Refinement on *F*^2^	Primary atom site location: structure-invariant direct methods
Least-squares matrix: full	Secondary atom site location: difference Fourier map
*R*[*F*^2^ > 2σ(*F*^2^)] = 0.035	Hydrogen site location: inferred from neighbouring sites
*wR*(*F*^2^) = 0.088	H-atom parameters constrained
*S* = 0.92	*w* = 1/[σ^2^(*F*_o_^2^) + (0.0518*P*)^2^] where *P* = (*F*_o_^2^ + 2*F*_c_^2^)/3
3735 reflections	(Δ/σ)_max_ = 0.001
299 parameters	Δρ_max_ = 0.46 e Å^−^^3^
0 restraints	Δρ_min_ = −0.40 e Å^−^^3^

### Special details

**Table:**

Geometry. All esds (except the esd in the dihedral angle between two l.s. planes) are estimated using the full covariance matrix. The cell esds are taken into account individually in the estimation of esds in distances, angles and torsion angles; correlations between esds in cell parameters are only used when they are defined by crystal symmetry. An approximate (isotropic) treatment of cell esds is used for estimating esds involving l.s. planes.

### Fractional atomic coordinates and isotropic or equivalent isotropic displacement parameters (Å^2^)

**Table:**

	*x*	*y*	*z*	*U*_iso_*/*U*_eq_	
C1	0.5650 (3)	0.70124 (15)	0.5969 (4)	0.0507 (8)	
H1	0.5048	0.7209	0.6227	0.061*	
C2	0.6310 (3)	0.73723 (16)	0.5286 (4)	0.0575 (9)	
H2	0.6170	0.7818	0.5098	0.069*	
C3	0.7204 (3)	0.70832 (18)	0.4856 (4)	0.0581 (9)	
H3	0.7639	0.7337	0.4355	0.070*	
C4	0.7451 (3)	0.64367 (16)	0.5157 (3)	0.0517 (8)	
H4	0.8048	0.6252	0.4860	0.062*	
C5	0.6664 (3)	0.43392 (15)	0.7299 (4)	0.0532 (8)	
H5	0.7314	0.4166	0.7140	0.064*	
C6	0.5970 (3)	0.39546 (15)	0.7872 (4)	0.0586 (9)	
H6	0.6154	0.3516	0.8099	0.070*	
C7	0.4988 (3)	0.41906 (15)	0.8135 (4)	0.0525 (8)	
H7	0.4519	0.3910	0.8503	0.063*	
C8	0.4724 (2)	0.48279 (14)	0.7851 (3)	0.0457 (7)	
H8	0.4078	0.4988	0.8052	0.055*	
C9	0.5181 (2)	0.59299 (13)	0.6949 (3)	0.0355 (7)	
N10	0.70556 (19)	0.54005 (11)	0.6297 (2)	0.0397 (6)	
C11	0.5865 (2)	0.63282 (13)	0.6305 (3)	0.0379 (7)	
C12	0.6800 (2)	0.60492 (14)	0.5919 (3)	0.0405 (7)	
C13	0.5419 (2)	0.52598 (13)	0.7246 (3)	0.0366 (7)	
C14	0.6396 (2)	0.50086 (13)	0.6942 (3)	0.0380 (7)	
C15	0.4213 (2)	0.62290 (13)	0.7426 (3)	0.0388 (7)	
O16	0.32648 (15)	0.60104 (8)	0.6463 (2)	0.0410 (5)	
O17	0.42777 (16)	0.66151 (10)	0.8497 (2)	0.0541 (6)	
C18	0.2280 (2)	0.62507 (14)	0.6773 (3)	0.0417 (7)	
C19	0.1815 (2)	0.68158 (14)	0.6025 (3)	0.0457 (8)	
C20	0.0812 (3)	0.70147 (17)	0.6251 (4)	0.0596 (9)	
H20	0.0476	0.7392	0.5743	0.072*	
C21	0.0311 (3)	0.6654 (2)	0.7228 (4)	0.0664 (10)	
H21	−0.0362	0.6791	0.7382	0.080*	
C22	0.0792 (3)	0.60940 (19)	0.7976 (4)	0.0637 (10)	
H22	0.0449	0.5855	0.8639	0.076*	
C23	0.1787 (3)	0.58855 (15)	0.7748 (4)	0.0509 (8)	
H23	0.2117	0.5505	0.8245	0.061*	
Br24	0.25462 (3)	0.731793 (18)	0.47628 (4)	0.06770 (16)	
C25	0.8105 (3)	0.51466 (16)	0.6062 (4)	0.0605 (9)	
H25A	0.8261	0.4728	0.6587	0.091*	
H25B	0.8685	0.5449	0.6522	0.091*	
H25C	0.8050	0.5098	0.4923	0.091*	
S26	0.17372 (8)	0.40673 (5)	0.95720 (10)	0.0608 (3)	
O27	0.2606 (2)	0.41546 (17)	0.8843 (4)	0.1142 (11)	
O28	0.1816 (3)	0.35388 (13)	1.0698 (3)	0.0968 (9)	
O29	0.1332 (2)	0.46570 (12)	1.0126 (3)	0.0852 (8)	
C30	0.0621 (3)	0.38096 (18)	0.7915 (4)	0.0625 (9)	
F31	0.0855 (2)	0.32387 (11)	0.7309 (3)	0.0976 (7)	
F32	0.0450 (2)	0.42450 (12)	0.6698 (2)	0.0985 (7)	
F33	−0.03080 (19)	0.37394 (13)	0.8308 (3)	0.1006 (8)	

### Atomic displacement parameters (Å^2^)

**Table:**

	*U*^11^	*U*^22^	*U*^33^	*U*^12^	*U*^13^	*U*^23^
C1	0.044 (2)	0.0501 (19)	0.0606 (18)	0.0065 (16)	0.0176 (16)	0.0073 (15)
C2	0.056 (2)	0.0465 (18)	0.072 (2)	−0.0008 (17)	0.0180 (19)	0.0134 (16)
C3	0.053 (2)	0.063 (2)	0.061 (2)	−0.0112 (18)	0.0200 (17)	0.0060 (16)
C4	0.045 (2)	0.060 (2)	0.0563 (18)	−0.0036 (17)	0.0233 (16)	−0.0003 (15)
C5	0.053 (2)	0.0488 (19)	0.0603 (19)	0.0127 (17)	0.0193 (16)	−0.0009 (15)
C6	0.068 (3)	0.0403 (18)	0.068 (2)	0.0100 (18)	0.0181 (19)	0.0076 (15)
C7	0.051 (2)	0.050 (2)	0.0576 (19)	−0.0015 (17)	0.0163 (16)	0.0083 (15)
C8	0.0402 (19)	0.0512 (19)	0.0463 (16)	0.0000 (15)	0.0122 (14)	0.0017 (14)
C9	0.0288 (16)	0.0437 (17)	0.0319 (13)	0.0022 (13)	0.0035 (12)	−0.0038 (12)
N10	0.0327 (14)	0.0451 (14)	0.0426 (13)	0.0004 (12)	0.0120 (11)	−0.0088 (11)
C11	0.0318 (17)	0.0444 (17)	0.0365 (14)	−0.0006 (14)	0.0065 (13)	0.0004 (12)
C12	0.0351 (18)	0.0513 (19)	0.0350 (14)	−0.0040 (15)	0.0086 (13)	−0.0073 (13)
C13	0.0297 (17)	0.0463 (18)	0.0328 (14)	0.0000 (13)	0.0058 (12)	−0.0044 (12)
C14	0.0345 (18)	0.0438 (18)	0.0342 (14)	0.0004 (14)	0.0056 (13)	−0.0061 (12)
C15	0.0392 (19)	0.0388 (16)	0.0392 (16)	0.0002 (14)	0.0112 (14)	0.0036 (13)
O16	0.0297 (11)	0.0477 (11)	0.0461 (11)	0.0011 (9)	0.0102 (9)	−0.0073 (9)
O17	0.0409 (13)	0.0674 (14)	0.0534 (12)	−0.0011 (11)	0.0105 (10)	−0.0193 (11)
C18	0.0297 (17)	0.0493 (18)	0.0453 (15)	0.0000 (15)	0.0079 (13)	−0.0118 (14)
C19	0.0331 (19)	0.0549 (19)	0.0455 (16)	0.0011 (15)	0.0031 (14)	−0.0113 (14)
C20	0.045 (2)	0.065 (2)	0.062 (2)	0.0092 (19)	−0.0010 (17)	−0.0164 (18)
C21	0.032 (2)	0.091 (3)	0.076 (2)	−0.001 (2)	0.0114 (19)	−0.033 (2)
C22	0.048 (2)	0.085 (3)	0.062 (2)	−0.016 (2)	0.0208 (18)	−0.0191 (19)
C23	0.042 (2)	0.0555 (19)	0.0585 (19)	−0.0048 (16)	0.0189 (16)	−0.0061 (15)
Br24	0.0705 (3)	0.0676 (3)	0.0633 (2)	0.01066 (19)	0.01349 (18)	0.01921 (17)
C25	0.045 (2)	0.058 (2)	0.086 (2)	0.0032 (17)	0.0298 (18)	−0.0099 (18)
S26	0.0498 (6)	0.0710 (6)	0.0608 (5)	−0.0004 (4)	0.0121 (4)	−0.0092 (4)
O27	0.0577 (19)	0.157 (3)	0.143 (3)	−0.0291 (19)	0.0528 (18)	−0.044 (2)
O28	0.130 (3)	0.0818 (18)	0.0673 (15)	0.0238 (17)	0.0033 (16)	0.0126 (14)
O29	0.108 (2)	0.0632 (16)	0.0888 (17)	0.0013 (15)	0.0326 (16)	−0.0232 (13)
C30	0.060 (3)	0.074 (3)	0.061 (2)	−0.008 (2)	0.0304 (19)	−0.0021 (19)
F31	0.123 (2)	0.0832 (16)	0.0873 (14)	−0.0155 (14)	0.0280 (14)	−0.0331 (12)
F32	0.1056 (19)	0.1177 (19)	0.0672 (13)	0.0148 (15)	0.0119 (13)	0.0236 (13)
F33	0.0587 (15)	0.140 (2)	0.1082 (17)	−0.0275 (14)	0.0301 (13)	−0.0134 (15)

### Geometric parameters (Å, º)

**Table:**

C1—C2	1.343 (4)	C13—C14	1.412 (4)
C1—C11	1.434 (4)	C15—O17	1.192 (3)
C1—H1	0.9300	C15—O16	1.342 (3)
C2—C3	1.397 (5)	O16—C18	1.417 (3)
C2—H2	0.9300	C18—C23	1.372 (4)
C3—C4	1.362 (5)	C18—C19	1.373 (4)
C3—H3	0.9300	C19—C20	1.383 (4)
C4—C12	1.407 (4)	C19—Br24	1.882 (3)
C4—H4	0.9300	C20—C21	1.375 (5)
C5—C6	1.351 (4)	C20—H20	0.9300
C5—C14	1.419 (4)	C21—C22	1.371 (5)
C5—H5	0.9300	C21—H21	0.9300
C6—C7	1.394 (4)	C22—C23	1.380 (5)
C6—H6	0.9300	C22—H22	0.9300
C7—C8	1.346 (4)	C23—H23	0.9300
C7—H7	0.9300	C25—H25A	0.9600
C8—C13	1.424 (4)	C25—H25B	0.9600
C8—H8	0.9300	C25—H25C	0.9600
C9—C11	1.391 (4)	S26—O27	1.397 (3)
C9—C13	1.406 (4)	S26—O28	1.428 (3)
C9—C15	1.505 (4)	S26—O29	1.430 (2)
N10—C14	1.362 (3)	S26—C30	1.801 (4)
N10—C12	1.378 (3)	C30—F33	1.301 (4)
N10—C25	1.476 (4)	C30—F31	1.334 (4)
C11—C12	1.416 (4)	C30—F32	1.340 (4)
			
C2—C1—C11	120.7 (3)	C13—C14—C5	118.7 (3)
C2—C1—H1	119.7	O17—C15—O16	124.5 (3)
C11—C1—H1	119.7	O17—C15—C9	124.6 (3)
C1—C2—C3	120.5 (3)	O16—C15—C9	110.8 (2)
C1—C2—H2	119.7	C15—O16—C18	117.1 (2)
C3—C2—H2	119.7	C23—C18—C19	122.0 (3)
C4—C3—C2	121.2 (3)	C23—C18—O16	118.3 (3)
C4—C3—H3	119.4	C19—C18—O16	119.6 (3)
C2—C3—H3	119.4	C18—C19—C20	118.6 (3)
C3—C4—C12	119.8 (3)	C18—C19—Br24	120.5 (2)
C3—C4—H4	120.1	C20—C19—Br24	120.9 (3)
C12—C4—H4	120.1	C21—C20—C19	120.0 (3)
C6—C5—C14	119.6 (3)	C21—C20—H20	120.0
C6—C5—H5	120.2	C19—C20—H20	120.0
C14—C5—H5	120.2	C22—C21—C20	120.7 (3)
C5—C6—C7	122.4 (3)	C22—C21—H21	119.7
C5—C6—H6	118.8	C20—C21—H21	119.7
C7—C6—H6	118.8	C21—C22—C23	120.0 (3)
C8—C7—C6	119.5 (3)	C21—C22—H22	120.0
C8—C7—H7	120.3	C23—C22—H22	120.0
C6—C7—H7	120.3	C18—C23—C22	118.8 (3)
C7—C8—C13	120.9 (3)	C18—C23—H23	120.6
C7—C8—H8	119.5	C22—C23—H23	120.6
C13—C8—H8	119.5	N10—C25—H25A	109.5
C11—C9—C13	120.8 (3)	N10—C25—H25B	109.5
C11—C9—C15	119.5 (2)	H25A—C25—H25B	109.5
C13—C9—C15	119.6 (3)	N10—C25—H25C	109.5
C14—N10—C12	121.7 (2)	H25A—C25—H25C	109.5
C14—N10—C25	120.3 (2)	H25B—C25—H25C	109.5
C12—N10—C25	117.9 (2)	O27—S26—O28	117.6 (2)
C9—C11—C12	119.2 (3)	O27—S26—O29	115.00 (18)
C9—C11—C1	122.7 (3)	O28—S26—O29	112.46 (17)
C12—C11—C1	118.0 (3)	O27—S26—C30	103.37 (18)
N10—C12—C4	121.1 (3)	O28—S26—C30	102.49 (17)
N10—C12—C11	119.3 (3)	O29—S26—C30	103.38 (17)
C4—C12—C11	119.6 (3)	F33—C30—F31	107.6 (3)
C9—C13—C14	118.3 (3)	F33—C30—F32	106.9 (3)
C9—C13—C8	122.8 (3)	F31—C30—F32	106.6 (3)
C14—C13—C8	118.9 (3)	F33—C30—S26	113.8 (2)
N10—C14—C13	120.5 (2)	F31—C30—S26	110.6 (3)
N10—C14—C5	120.9 (3)	F32—C30—S26	110.8 (2)
			
C11—C1—C2—C3	1.5 (5)	C8—C13—C14—N10	177.1 (2)
C1—C2—C3—C4	−1.9 (5)	C9—C13—C14—C5	177.5 (2)
C2—C3—C4—C12	−0.2 (4)	C8—C13—C14—C5	−2.0 (4)
C14—C5—C6—C7	−0.1 (5)	C6—C5—C14—N10	−177.1 (3)
C5—C6—C7—C8	−1.7 (5)	C6—C5—C14—C13	2.0 (4)
C6—C7—C8—C13	1.6 (4)	C11—C9—C15—O17	−65.8 (3)
C13—C9—C11—C12	1.2 (4)	C13—C9—C15—O17	110.5 (3)
C15—C9—C11—C12	177.5 (2)	C11—C9—C15—O16	113.1 (3)
C13—C9—C11—C1	179.5 (2)	C13—C9—C15—O16	−70.5 (3)
C15—C9—C11—C1	−4.3 (4)	O17—C15—O16—C18	−1.1 (4)
C2—C1—C11—C9	−177.3 (3)	C9—C15—O16—C18	179.9 (2)
C2—C1—C11—C12	1.0 (4)	C15—O16—C18—C23	−94.4 (3)
C14—N10—C12—C4	−175.8 (2)	C15—O16—C18—C19	89.3 (3)
C25—N10—C12—C4	7.3 (4)	C23—C18—C19—C20	−0.9 (4)
C14—N10—C12—C11	4.1 (3)	O16—C18—C19—C20	175.3 (2)
C25—N10—C12—C11	−172.7 (2)	C23—C18—C19—Br24	177.7 (2)
C3—C4—C12—N10	−177.4 (3)	O16—C18—C19—Br24	−6.2 (3)
C3—C4—C12—C11	2.7 (4)	C18—C19—C20—C21	1.0 (4)
C9—C11—C12—N10	−4.6 (3)	Br24—C19—C20—C21	−177.5 (2)
C1—C11—C12—N10	177.0 (2)	C19—C20—C21—C22	−0.4 (5)
C9—C11—C12—C4	175.3 (2)	C20—C21—C22—C23	−0.3 (5)
C1—C11—C12—C4	−3.0 (4)	C19—C18—C23—C22	0.2 (4)
C11—C9—C13—C14	2.7 (3)	O16—C18—C23—C22	−176.0 (2)
C15—C9—C13—C14	−173.5 (2)	C21—C22—C23—C18	0.4 (4)
C11—C9—C13—C8	−177.8 (2)	O27—S26—C30—F33	−175.8 (3)
C15—C9—C13—C8	6.0 (4)	O28—S26—C30—F33	61.5 (3)
C7—C8—C13—C9	−179.3 (2)	O29—S26—C30—F33	−55.6 (3)
C7—C8—C13—C14	0.2 (4)	O27—S26—C30—F31	62.8 (3)
C12—N10—C14—C13	0.0 (3)	O28—S26—C30—F31	−59.9 (3)
C25—N10—C14—C13	176.7 (2)	O29—S26—C30—F31	−177.0 (2)
C12—N10—C14—C5	179.0 (2)	O27—S26—C30—F32	−55.2 (3)
C25—N10—C14—C5	−4.2 (4)	O28—S26—C30—F32	−177.9 (3)
C9—C13—C14—N10	−3.4 (3)	O29—S26—C30—F32	65.0 (3)

### Hydrogen-bond geometry (Å, º)

**Table:**

*D*—H···*A*	*D*—H	H···*A*	*D*···*A*	*D*—H···*A*
C3—H3···O28^i^	0.93	2.54	3.289 (5)	137
C7—H7···O27	0.93	2.54	3.200 (5)	128

Symmetry code: (i) −*x*+1, *y*+1/2, −*z*+3/2.

### C—F···π, C—Br···π and S—O···π interactions (Å, °).

*Cg*1, *Cg*2 and *Cg*4 are the centroids of the C9/N10/C11–C14, C1–C4/C11/C12 and C18–C23 rings, respectively.

**Table:**

*X*—*I*···*J*	*I*···*J*	*X*···*J*	*X*—*I*···*J*
C19—Br24···*Cg*4^ii^	3.523 (2)	4.847 (3)	124.6 (1)
C30—F32···*Cg*4^iii^	3.648 (2)	4.310 (4)	110.8 (2)
S26—O27···*Cg*1^iv^	3.821 (3)	3.708 (2)	74.8 (2)
S26—O28···*Cg*1^iv^	3.414 (3)	3.708 (2)	90.3 (2)
S26—O28···*Cg*2^iv^	3.358 (3)	4.445 (2)	132.2 (2)

Symmetry codes: (ii) *x*, –*y* + 3/2, *z* – 1/2; (iii) –*x*, –*y* + 1, –*z* + 1; (iv) –*x* + 1, –*y* + 1, –*z* + 2.

### π–π interactions (Å, °).

*Cg*1 and *Cg*3 are the centroids of the C9/N10/C11–C14 and C5–C8/C13/C14 rings, respectively. Cg*I*···Cg*J* is the distance between ring centroids. The dihedral angle is that between the planes of the rings *I* and *J*. *CgI*_Perp is the perpendicular distance of *CgI* from ring *J*. Cg*I*_Offset is the distance between *CgI* and perpendicular projection of *CgJ* on ring *I*.

**Table:**

*I*	*J*	*CgI*···*CgJ*	Dihedral angle	*CgI*_Perp	Cg*I*_Offset
1	3^v^	3.744 (2)	2.74 (13)	3.703 (2)	0.553 (2)
3	1^v^	3.744 (2)	2.74 (13)	3.717 (2)	0.549 (2)

Symmetry code: (v) –*x* + 1, –*y* + 1, –*z* + 1.
